# Safety, Pharmacokinetics, and Efficacy of Olorinab, a Peripherally Acting, Highly Selective, Full Agonist of the Cannabinoid Receptor 2, in a Phase 2a Study of Patients With Chronic Abdominal Pain Associated With Crohn’s Disease

**DOI:** 10.1093/crocol/otaa089

**Published:** 2020-10-24

**Authors:** Bruce R Yacyshyn, Stephen Hanauer, Preston Klassen, Brett A English, Kathe Stauber, Charles F Barish, Kye Gilder, Stewart Turner, Peter D R Higgins

**Affiliations:** 1 Department of Gastroenterology, University of Cincinnati, Cincinnati, Ohio, USA; 2 Department of Gastroenterology and Hepatology, Northwestern University, Chicago, Illinois, USA; 3 Arena Pharmaceuticals Inc., Research and Development, San Diego, California, USA; 4 Arena Pharmaceuticals Inc., Clinical Development, San Diego, California, USA; 5 Arena Pharmaceuticals Inc., Nonclinical Development and Clinical Pharmacology, San Diego, California, USA; 6 Department of Gastroenterology, University of North Carolina School of Medicine, Chapel Hill, North Carolina, USA; 7 Arena Pharmaceuticals Inc., Biostatistics and Data Management, San Diego, California, USA; 8 Arena Pharmaceuticals Inc., Olorinab Global Program Lead, San Diego, California, USA; 9 Department of Gastroenterology, University of Michigan, Ann Arbor, Michigan, USA

**Keywords:** Crohn’s disease, cannabinoid, CB_2_ agonist, olorinab, pain

## Abstract

**Background:**

This randomized, open-label phase 2a study investigated the safety/tolerability, pharmacokinetics, and efficacy of olorinab—a highly selective, peripherally acting, full agonist of the cannabinoid receptor 2—in patients with Crohn’s disease (CD) experiencing abdominal pain.

**Methods:**

Eligible subjects 18–80 years of age with quiescent to mildly active CD were randomized to receive olorinab 25 or 100 mg three times daily for 8 weeks. The primary objective was to assess safety/tolerability.

**Results:**

Fourteen subjects received olorinab 25 mg (N = 6) or 100 mg (N = 8). Ten subjects [4 (67%) in the 25-mg group and 6 (75%) in the 100-mg group] reported a total of 34 treatment-emergent adverse events (TEAEs; 32 grade 1/2, not serious events; 2 grade 3, serious, not treatment-related events). No dose reductions or discontinuations due to TEAEs or deaths were reported. Dose-proportional increases in olorinab exposure from 25 to 100 mg were observed, with minimal accumulation at both doses. At week 8, the mean (SD) change from baseline in average abdominal pain score at peak olorinab plasma concentrations was −4.61 (1.77) in the 25-mg group (*P* = 0.0043) and −4.57 (2.17) in the 100-mg group (*P* = 0.0036). The change from baseline at week 8 in the mean (SD) number of pain-free days per week was +1.60 (2.61) in the 25-mg group and +2.33 (3.62) in the 100-mg group. No subject required pain medication on study.

**Conclusions:**

Patients with quiescent to mildly active CD receiving olorinab experienced mild-to-moderate adverse events and an improvement in abdominal pain scores in this study.

## INTRODUCTION

Crohn’s disease (CD) is a chronic inflammatory disease of the gastrointestinal tract with heterogenous symptoms, often including abdominal pain and diarrhea, that evolve in a relapsing and remitting manner.^1^ Abdominal pain is commonly reported in patients with CD and is associated with reduced quality of life.^2^ Even with apparent remission of inflammation, approximately 41% of patients with CD still experience irritable bowel syndrome-like symptoms, including abdominal pain, bloating, or erratic bowel habits.^3^ In many cases, abdominal pain is severe enough to warrant pain-specific treatment, but current treatment options are limited. Approaches to abdominal pain management in patients with CD include analgesics (eg, acetaminophen), nonsteroidal anti-inflammatory drugs (NSAIDs; eg, ibuprofen and naproxen), antispasmodics (hyoscyamine, dicyclomine), antidepressants (eg, selective serotonin reuptake inhibitors, tricyclic antidepressants), and opioids, but these strategies have demonstrated limited efficacy and/or unfavorable adverse event (AE) profiles.^4^ For example, patients receiving opioids have the potential for developing tolerance, addiction and abuse, and respiratory depression and death,^5^ whereas NSAIDs are associated with an increased risk of gut mucosal damage, ulceration, and induction of irritable bowel disease flares.^6^ Given these challenges, there is a significant unmet need for a novel pharmacologic approach to treat abdominal pain in patients with CD.

Targeted cannabinoid receptor agonists may be an attractive treatment for abdominal pain without the potential for disease exacerbation,^7^ and cannabis has demonstrated promising results in the treatment of pain associated with CD.^8,9^ There are 2 known cannabinoid receptors: cannabinoid receptor 1 (CB_1_) and cannabinoid receptor 2 (CB_2_). Under physiological conditions, CB_2_ is expressed on gastrointestinal enteric nerves, select immune cells, and on healthy colonic epithelium.^10–12^ CB_2_ expression is increased in the ulcerative margin in CD,^10^ and CB_2_ has been shown to be upregulated in the gastrointestinal tract during intestinal inflammation and to modulate visceral sensitivity in animal models.^7^

Research in human explant tissue has shown that CB_2_, but not CB_1_, may protect against the cytokine-mediated inflammation and epithelial damage that are known to contribute to abdominal pain.^13^ CB_2_ knockout mice have demonstrated enhanced colitis induction, supporting the potential protective effects of CB_2_ activation.^14^ In animal models of chemically induced visceral hypersensitivity, CB_2_ activation had an analgesic effect.^15,16^ Taking all this into account, agonists of CB_2_ may have the potential to provide pain relief in patients with CD experiencing abdominal pain without the off-target effects of cannabis, making CB_2_ an attractive therapeutic target.

Previous cannabinoid receptor agonists have shown preclinical efficacy for pain.^17^ However, the therapeutic utility of nonselective, brain-penetrating cannabinoid receptor agonists has been limited by undesirable psychoactive effects associated with the lack of selectivity for CB_2_ resulting in activation of CB_1_,^17^ or potentially through incomplete agonism of CB_2_ leading to receptor desensitization and tachyphylaxis.^18^ Selective targeting of CB_2_ may alleviate abdominal pain, as suggested by a preclinical study of a CB_2_ agonist that blocked mesenteric nerve firing,^19^ and by the ability of a CB_2_ antagonist to block probiotic-induced intestinal analgesia in a butyrate-induced model of colonic hypersensitivity.^20^

Olorinab (APD371) is an oral, peripherally acting and highly selective, full agonist of CB_2_.^18^ Olorinab is a small molecule that has exhibited >1000-fold selectivity for CB_2_ over CB_1_ and shows minimal-to-no off-target activity for a broad range of other noncannabinoid receptors, ion channels, and transporters.^18^ Olorinab has been shown to activate endogenous CB_2_ in primary rat splenocytes, human HL-60 cells, and primary human B cells.^21^ Through activation of CB_2_, olorinab decreased the hypersensitivity of colonic nociceptors in mice with colitis (unpublished data) and reduced pain in several animal models,^21^ and olorinab has shown low brain penetration in rats.^18^ Olorinab is cleared mainly through biotransformation to multiple metabolites, with 3 of its main circulating metabolites (M1, M2, and M4) being much less potent—ranging from approximately 10- to 200-fold lower affinity for recombinant human CB_2_ than olorinab (unpublished data). Olorinab was generally safe and well tolerated in phase 1 studies of healthy volunteers.^22^ The present study assessed the safety and efficacy of olorinab in patients with abdominal pain associated with CD.

## MATERIALS AND METHODS

### Ethical Considerations

The trial was approved by the local and central Institutional Review Boards (IRBs), including Medical School IRB, Quorum Review IRB (now Advarra), and Northwestern University IRB, and was conducted in accordance with Good Clinical Practice Guidelines of the International Conference on Harmonisation and the principles of the Declaration of Helsinki. All subjects provided written informed consent.

### Study Design

This was an open-label, randomized, parallel-group, multicenter phase 2a study (ClinicalTrials.gov ID: NCT03155945). The study comprised a screening period of up to 4 weeks, an 8-week randomized treatment period, and a 2-week follow-up period. Eligible subjects were randomized (stratified by sex) in a 1:1 ratio to olorinab 25 or 100 mg three times daily (TID) for 8 weeks. Randomization was undertaken by assignment to an appropriate dose group and unique subject number from a centralized master list; all subjects were required to start treatment within 1 week of randomization.

### Subjects

The study enrolled adult subjects with moderate-to-severe abdominal pain [average abdominal pain score (AAPS) of ≥4 on 7 consecutive days during the screening period] due to quiescent to mildly active inflammatory CD (total simple endoscopic score for CD of <10 or fecal calprotectin of <500 µg/g within 4 weeks of screening). Eligible subjects were male or female, 18–80 years of age, inclusive, with a clinical diagnosis of endoscopically and histopathologically confirmed CD for at least 3 months prior to screening. Subjects receiving concomitant biologic or anti-inflammatory therapies for CD were required to be on a stable dose. No clinically significant abnormalities in physical examinations, laboratory findings, and 12-lead electrocardiograms were permitted. Key exclusion criteria were use of medical marijuana, tetrahydrocannabinol, or its derivatives during screening and study treatment period; evidence of abdominal abscess at the screening visit; subtotal or total colectomy; a permanent ostomy; history of >3 small bowel resections, diagnosis of short bowel syndrome, or bowel resection within 6 months prior to randomization; history or evidence of adenomatous colonic polyps or colonic mucosal dysplasia; diagnosis of indeterminate colitis, ulcerative colitis, or clinical findings suggestive of ulcerative colitis; evidence of current gastrointestinal infection (bacterial or parasitic) or significant infection within 45 days of screening; and clinically significant extraintestinal infection within 30 days of screening.

### Treatment

Olorinab capsules were self-administered, with no food restrictions, except for the exclusion of grapefruit products or prune juice. Laxatives were also restricted 1 day prior to randomization and throughout the treatment period. Olorinab 25 or 100 mg was scheduled to be taken TID (at approximately 07:00 ± 2, 15:00 ± 2, and 23:00 ± 2 hours for 8 weeks) on days 1–56.

### Study Endpoints and Assessments

The primary objective of this study was to assess the safety and tolerability of 2 different doses of olorinab in subjects with CD experiencing abdominal pain treated for up to 8 weeks. Key exploratory endpoints included pharmacokinetic (PK) profiles (including metabolites) and average PK parameters (maximum (peak) observed plasma concentration [*C*_max_], time to reach maximum (peak) observed plasma concentration [*t*_max_], area under the concentration–time curve from 0 to 8 hours postdose [AUC_0–8_]) of 2 doses of olorinab TID; change in abdominal pain score (APS) from predose (trough concentration) to 1.5 hours postdose (peak concentration) following the first of 3 daily doses of olorinab (assessed daily to day 56 and average weekly to week 8); change in AAPS from baseline to week 8 (averaged weekly to week 8); proportion of subjects who were pain relief responders (≥30% reduction from baseline in AAPS) weekly and at the end of treatment (EOT); number of pain-free days per week based on responses to the APS in each treatment group; pain medication use; changes in C-reactive protein (CRP) and fecal calprotectin at weeks 4 and 8; changes in CD patient-reported outcome (CD-PRO) domain scores from baseline to week 8; and changes in the Patient Health Questionnaire-9 (PHQ-9) scores at screening, week 4, and week 8. Other analyses included change in AAPS at the pre-evening dose (evening trough concentration) and change in weekly AAPS from baseline to week 4 of treatment.

Safety assessments included TEAEs (defined as an AE that occurred during study treatment); serious adverse events (SAEs); clinical laboratory tests; physical examinations; vital sign measurements; and 12-lead electrocardiograms. Blood samples for PK assessments were collected before the first daily dose and 0.5, 1, 2, 4, 6, 8 (prior to second daily dose), 9, 10, and 24 (prior to first daily dose on day 2) hours thereafter on day 1; prior to the first daily dose only during weeks 2, 4, and 6; and prior to the first daily dose and 0.5, 1, 2, 4, 6, 8 (prior to second daily dose), and 24 hours thereafter during week 8. Plasma concentrations of olorinab and its less-active metabolites M1, M2, and M4 were measured using a validated bioanalytical method. The method applied liquid–liquid sample extraction followed by liquid chromatography–tandem mass spectrometry detection. The lower limit of quantification for all analytes was 0.5 ng/mL. Abdominal pain was assessed using the APS and scored based on the 11-point numeric rating scale from 0 (no abdominal pain) to 10 (worst possible abdominal pain). APS was assessed during screening twice daily for at least 7 consecutive days (early morning and late evening) and TID during treatment (days 1–56; before the morning dose and any other study procedures; at 1.5 hours after the morning dose; and before the evening dose). A post hoc analysis of 6 derived CD-PRO domains (bowel, abdominal function, systemic symptoms, coping, daily impact, and emotional) was performed.

### Statistical Analysis

No formal sample size/power calculations and hypothesis testing were specified for this proof-of-concept study. Approximately 16 subjects were considered a reasonable sample size to assess the main study endpoints.

The Safety population included all randomized subjects who received at least 1 dose of olorinab; analyses of all safety variables use the Safety population. The Pharmacokinetic population included all randomized subjects who received at least 1 dose of olorinab and had at least 1 evaluable plasma concentration–time profiles. PK parameters were calculated using noncompartmental analysis. The Efficacy population included all randomized subjects who received at least 1 dose of olorinab and completed at least 7 days of assessments of APS on treatment up to week 4, and analyses of all efficacy variables use the Efficacy population. For the pain relief responder analysis, the primary analysis approach was nonresponder imputation, in which subjects who withdrew early or did not have week 8 data were considered nonresponders. Two sensitivity responder analyses were conducted: observed data analysis (using observed data only) and EOT (equivalent to last-observation-carried-forward) analysis, in which the last available value was used for subjects who withdrew early or did not have week 8 data. Noninferential between-dose cohort comparisons for the main study endpoint measures were performed using parametric or nonparametric methods as appropriate and based on a 2-sided hypothesis test at the 0.05 level of significance. All statistical analyses were performed using SAS software (Version 9.4, SAS Institute Inc., Cary, NC).

## RESULTS

### Subjects

A total of 14 subjects were randomized to treatment with olorinab 25 mg TID (N = 6) or 100 mg TID (N = 8). Demographic and baseline characteristics were comparable between the 2 treatment groups (Table 1). Of the 14 subjects enrolled in the study, corticosteroids were used prior to study entry in 6/14 (42.9%) subjects. Eleven subjects completed the study, which included the follow-up visit at week 10. Three subjects in the olorinab 100-mg group did not complete the study; reasons included lost to follow-up, withdrawal of consent, and other (n = 1 each). The median (range) duration of treatment was 8.2 (8–10) weeks for olorinab 25 mg and 8.1 (4–9) weeks for olorinab 100 mg. In the olorinab 25- and 100-mg groups, the median average dose per day was 72.7 and 289.6 mg, respectively. Subject compliance was high with 100% of subjects achieving at least 80% compliance.

**Table 1.** Demographics and Baseline Characteristics

	Olorinab 25 mg TID (N = 6)	Olorinab 100 mg TID (N = 8)	All Subjects (N = 14)
Age, mean (SD), years	35.0 (10.8)	36.9 (15.2)	36.1 (13.1)
Female, n (%)	4 (66.7)	4 (50.0)	8 (57.1)
Race, n (%)			
White	5 (83.3)	7 (87.5)	12 (85.7)
Black or African American	0	1 (12.5)	1 (7.1)
American Indian or Alaskan Native	1 (16.7)	0	1 (7.1)
Weight, mean (SD), kg	82.9 (17.8)	87.8 (22.3)	85.7 (19.9)
BMI, mean (SD), kg/m^2^	30.8 (7.7)	29.2 (5.7)	29.9 (6.4)
Nicotine use, n (%)			
Never	5 (83.3)	7 (87.5)	12 (85.7)
Former	1 (16.7)	1 (12.5)	2 (14.3)
Current	0	0	0
Alcohol use, n (%)			
Never	4 (66.7)	5 (62.5)	9 (64.3)
Former	0	0	0
Current	2 (33.3)	3 (37.5)	5 (35.7)
Time since CD diagnosis at screening, mean (SD), years	15.0 (6.4)	8.8 (8.9)	11.4 (8.3)
Location of CD, n (%)			
Small intestine	3 (50.0)	7 (87.5)	10 (71.4)
Colon	4 (66.7)	5 (62.5)	9 (64.3)
Rectum	1 (16.7)	2 (25.0)	3 (21.4)
Perianal	1 (16.7)	2 (25.0)	3 (21.4)
Baseline AAPS, mean (SD)	5.8 (1.3)	5.5 (2.0)	5.6 (1.7)
Patients receiving concomitant medications for CD, n (%)			
Azathioprine	0	4 (50.0)	4 (28.6)
Adalimumab	1 (16.7)	2 (25.0)	3 (21.4)
Infliximab	2 (33.3)	1 (12.5)	3 (21.4)
Mesalazine	1 (16.7)	1 (12.5)	2 (14.3)
Prednisone	1 (16.7)	1 (12.5)	2 (14.3)
Methotrexate	1 (16.7)	0	1 (7.1)
Ustekinumab	0	1 (12.5)	1 (7.1)
Vedolizumab	1 (16.7)	0	1 (7.1)

BMI, body mass index.

### Safety and Tolerability

Thirty-four TEAEs occurred in subjects treated with olorinab during this study. TEAEs were reported by 10 subjects—4 subjects (67%) and 6 subjects (75%) in the olorinab 25- and 100-mg TID groups, respectively (Table 2). These AEs were generally mild-to-moderate with only 2 grade 3 AEs occurring in 1 subject in the 100-mg group. The 2 grade 3 AEs (interstitial lung disease and acute interstitial pneumonitis) were reported by the investigator as SAEs but not related to study treatment. This same subject reported a total of 20 out of the 34 TEAEs that occurred in this study. Of the 34 TEAEs, only 1 TEAE was considered related to study treatment (grade 2 headache) as determined by the investigator. Twenty-seven of the 34 TEAEs had resolved and 7 of the 34 TEAEs were resolving or not resolved at the time of study conclusion.

**Table 2.** Summary of TEAEs

	Olorinab 25 mg TID (N = 6)	Olorinab 100 mg TID (N = 8)	All Subjects (N = 14)
No. of TEAEs	5	29	34
Subjects with ≥1 AE, n (%)	4 (66.7)	6 (75.0)	10 (71.4)
Subjects with ≥1 grade 3, n (%)* ^,†^	0	1 (12.5)	1 (7.1)
Subjects with ≥1 treatment-related AE, n (%)	0	1 (12.5)	1 (7.1)
AE preferred term reported by ≥2 subjects, n (%)			
Drug hypersensitivity	1 (16.7)	1 (12.5)	2 (14.3)
Hypomagnesemia	0	2 (25.0)	2 (14.3)
Pain in extremity	0	2 (25.0)	2 (14.3)
Subjects with ≥1 serious AE, n (%)^†^	0	1 (12.5)	1 (7.1)
Acute interstitial pneumonitis	0	1 (12.5)	1 (7.1)
Interstitial lung disease	0	1 (12.5)	1 (7.1)

Each subject is counted only once within each preferred term. AEs reflect those that occurred after the initial study dose of olorinab (ie, not during screening) and were coded using Medical Dictionary for Regulatory Activities, version 21.0.

*No subjects had a grade 4 or grade 5 TEAE.

^†^One subject receiving olorinab 100 mg TID reported 20 TEAEs, including 2 serious AEs (grade 3) that were considered not related to study treatment.

No treatment discontinuations, dose reductions, or dose interruptions due to AEs, grade 4 AEs, or deaths were reported. In addition, there were no clinically significant changes in vital signs or clinical safety laboratory findings were observed during the study.

### Pharmacokinetics

Plasma exposures to olorinab and its metabolites are shown in Supplementary Table 1. The median time to reach *C*_max_ (*t*_max_) was approximately 1–2 hours following a single dose (day 1) or repeat TID dosing (week 8) of olorinab 25 or 100 mg, indicating rapid absorption. Dose-proportional increases in olorinab *C*_max_ and AUC_0–8_ from 25 to 100 mg were observed following a single dose (day 1) and repeat TID dosing (week 8), with minimal accumulation at both dose levels. Among the olorinab metabolites, M1 *C*_max_ and AUC_0–8_ increased in a manner proportional to olorinab dose after a single dose and slightly greater than dose-proportional after repeated TID dosing, whereas M2 and M4 exposures increased less than dose-proportionally after a single dose and repeated TID dosing. M1 was the most predominant metabolite with week 8 exposures approximately 18%–64% lower than olorinab; M2 and M4 exposures were approximately 62%–91% lower than olorinab (Supplementary Table 2). Minimal plasma accumulation of M1 was observed at both dose levels, with the highest accumulation following 25 mg TID dosing; M2 and M4 accumulation could not be accurately determined due to their slow rate of formation.

### Key Exploratory Endpoints

**Figure 1.**
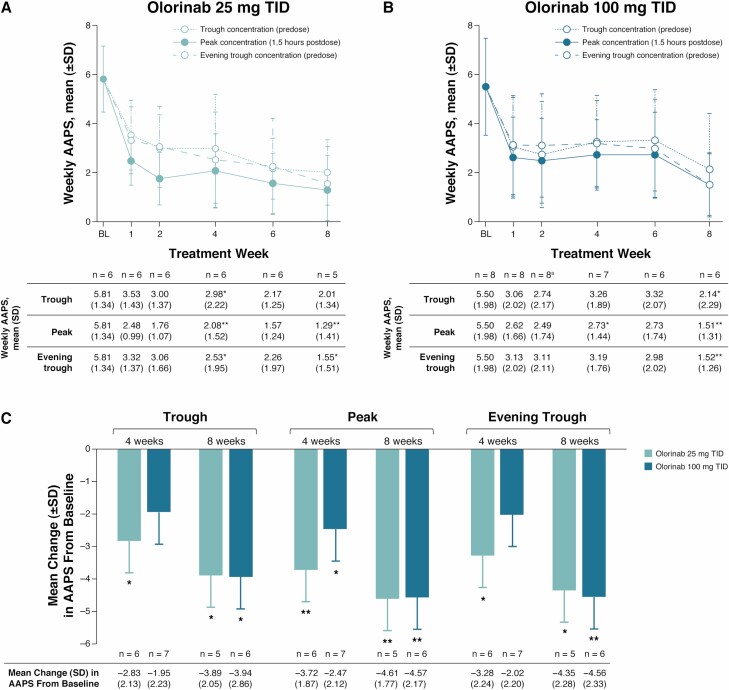
Change in weekly AAPS at trough, peak, and evening trough olorinab plasma concentrations. Mean change over time in weekly AAPS measured at trough (predose), peak (1.5 hours postdose), and evening trough (predose) olorinab plasma concentrations in the 25-mg (A) and 100-mg (B) TID dose groups. Summary of mean change from baseline in AAPS at weeks 4 and 8 in the olorinab 25- and 100-mg TID dose groups (C). ^a^For evening trough at week 2, n = 7 for mean weekly AAPS. *P < 0.05; **P < 0.01 vs baseline. BL, baseline.

Statistically significant improvements from baseline in AAPS were observed at trough, peak, and evening trough olorinab concentrations in both the 25- and 100-mg TID dose groups at week 8 of treatment (Fig. 1). At week 8, the mean (SD) change from baseline in AAPS measured at peak olorinab plasma concentrations was −4.61 (1.77) in the 25-mg dose group (*P* = 0.0043), −4.57 (2.17) in the 100-mg dose group (*P* = 0.0036), and −4.59 (1.90) in all subjects (*P* < 0.001). The mean change in AAPS from baseline to week 8 at trough and evening trough olorinab plasma concentrations was also significantly improved in both dose groups (all *P* < 0.05).

**Figure 2.**
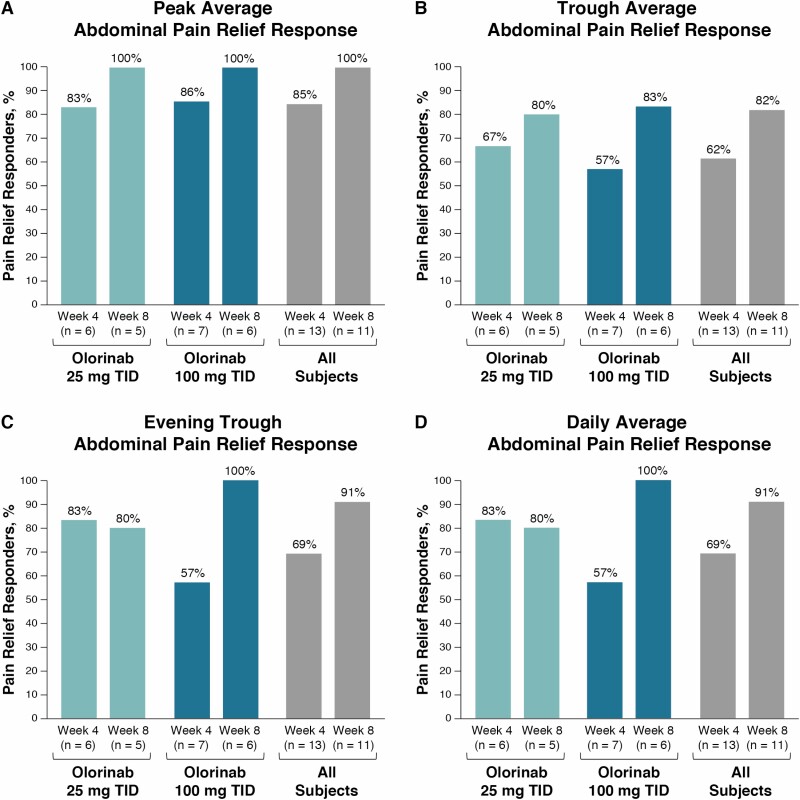
Pain relief response with olorinab. Proportion of patients who had ≥30% reduction from baseline in weekly peak AAPS (A), trough AAPS (B), evening trough AAPS (C), and daily AAPS (averaged over peak, trough, and evening trough diary entries; D) with olorinab 25 or 100 mg TID and in all subjects at weeks 4 and 8, and were considered pain relief responders. Pain relief response was assessed using observed data (ie, in subjects with evaluable data at weeks 4 and 8).

Pain relief response (defined as ≥30% reduction from baseline in weekly AAPS) in the overall population at peak olorinab plasma concentrations was achieved in most evaluable subjects at week 4 (85%) and all evaluable subjects at week 8 (100%) (Fig. 2A), with similar response rates observed in the 25-mg (83% and 100%) and the 100-mg (86% and 100%) groups at weeks 4 and 8, respectively. Pain relief response was also observed at trough and evening trough olorinab plasma concentrations and for daily average abdominal pain at week 4 (>50%) and week 8 (≥80%) (Figs. 2B–D). Using the nonresponder imputation analysis, most subjects (>60%) achieved peak, trough, evening trough, and daily average pain relief response at week 8, and results were similar using the EOT sensitivity analysis (Supplementary Fig. 1).

**Figure 3.**
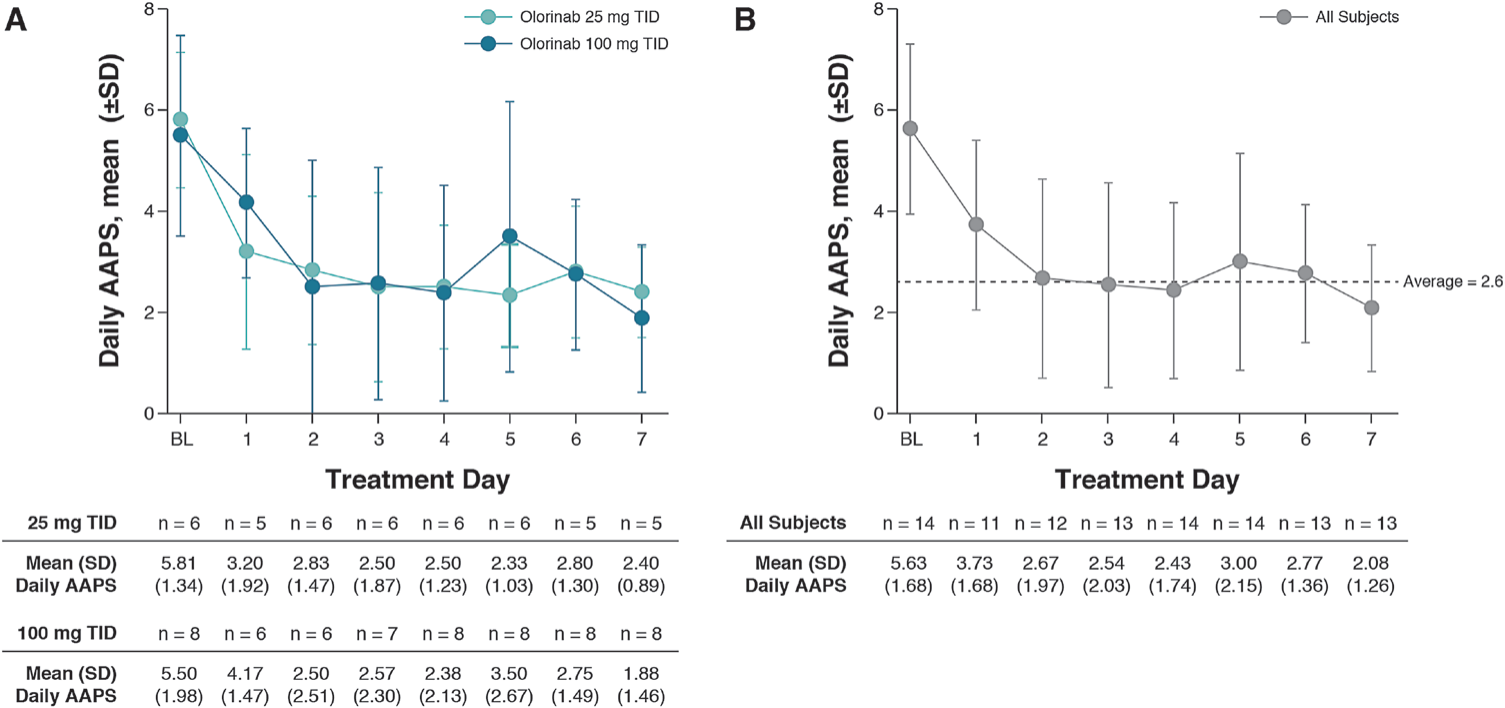
Daily AAPS in the first week of treatment with olorinab. Mean (SD) daily AAPS during the first week of treatment with olorinab 25 mg TID and olorinab 100 mg TID (A), and in all subjects (B). BL, baseline.

Among all subjects, mean daily AAPS was reduced from 5.63 at baseline to 2.67 within 2 days of treatment, and remained relatively stable for the rest of the first week of treatment (Fig. 3). The change from baseline in mean (SD) AAPS on day 1 of treatment was −0.63 (1.21) at trough, −2.02 (1.93) at peak, and −2.72 (2.48) at evening trough olorinab plasma concentrations in all subjects, with similar results seen in each dose group.

**Figure 4.**
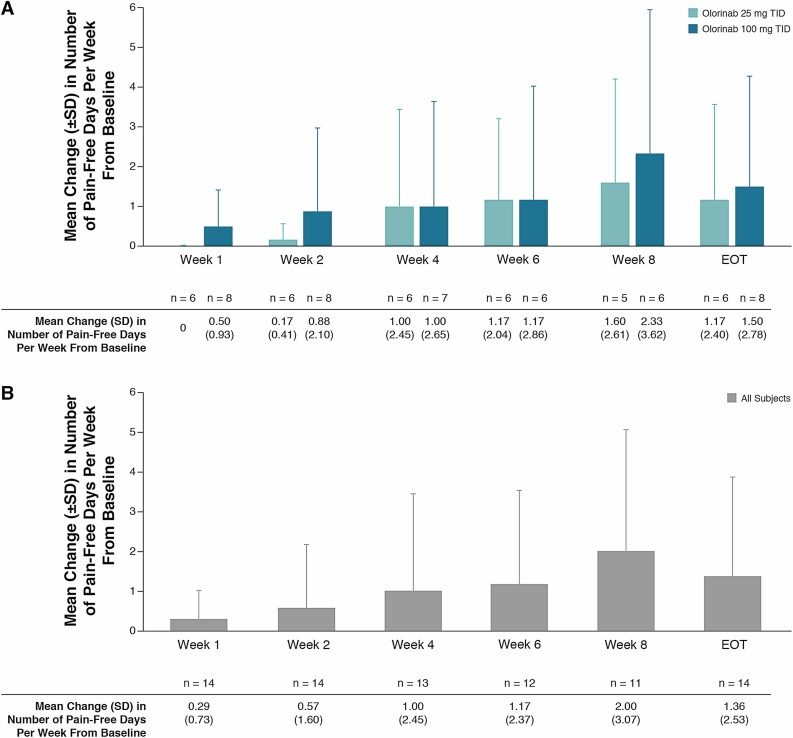
Change in the number of pain-free days per week with olorinab. Change from baseline in the mean (SD) number of pain-free days per week with olorinab 25 mg TID and olorinab 100 mg TID (A) and in all subjects (B). No subjects experienced a pain-free day at baseline. EOT analysis used the last available value for subjects who withdrew early or did not have week 8 data.

In the overall population, the change from baseline in the mean number of pain-free days per week increased from week 1 through week 8 (Fig. 4). There was an increase from baseline in the mean (SD) number of pain-free days per week in both the 25- and 100-mg TID dose groups at week 4 [1.00 (2.45) and 1.00 (2.65), respectively], week 8 [1.60 (2.61) and 2.33 (3.62)], and EOT [1.17 (2.40) and 1.50 (2.78)].

Pain medication was not required by any subject during the study. No significant changes from baseline in CRP or fecal calprotectin levels were observed at weeks 4 and 8 in either dose group (Supplementary Fig. 2).

### CD Patient-Reported Outcomes

**Figure 5.**
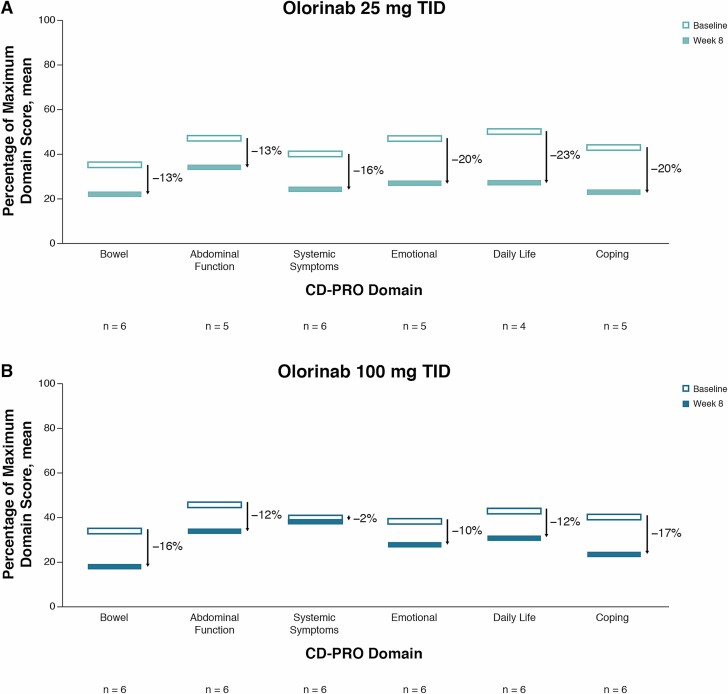
Percent change in mean CD-PRO domain scores with olorinab. Absolute percent change in mean CD-PRO scores with olorinab 25 mg TID (A) and olorinab 100 mg TID (B) across domains. Percent change was calculated separately for each domain by subtracting the mean week 8 score as a percentage of the maximum possible domain score from the baseline mean score as a percentage of the maximum possible domain score, for patients with both baseline and week 8 data.

Subjects in both dose groups reported significant improvements in the percent change from baseline in bowel domain scores at week 8 (both *P* < 0.05); the 25-mg dose group also reported significant improvements in the percent change from baseline in systemic symptoms, coping, daily impact, and emotional domain scores (all *P* < 0.05), and the 100-mg dose group reported a significant improvement in percent change from baseline in the abdominal function domain score (*P* < 0.05) (Supplementary Table 3). In the overall study population, percent changes from baseline in all CD-PRO domain scores (with the exception of systemic symptoms) were significantly improved at week 8 (*P* < 0.05). Similarly, the change from baseline in PHQ-9 score in the overall study population was significantly improved at week 4 (*P* = 0.005) and week 8 (*P* = 0.034). The overall improvement for each CD-PRO domain score from baseline to week 8 when expressed as percentages of the maximum possible domain score is shown in Fig. 5.

## DISCUSSION

This phase 2a study evaluating 2 doses (25 and 100 mg TID) of olorinab during an 8-week period demonstrated that this highly selective, peripherally acting, full agonist of CB_2_ was generally safe and well tolerated in patients with quiescent to mildly active CD. Most AEs observed with olorinab were mild-to-moderate in severity, with only 2 grade 3, SAEs reported in a single subject (neither considered to be related to study treatment). Furthermore, no central nervous system (CNS)-related AEs were reported with olorinab, in contrast to the frequent CNS effects seen with nonselective cannabinoids.^23^ This study also provided preliminary evidence of olorinab efficacy in improving APSs in this patient population. CB_2_ has received significant attention as a potential target that may provide pain relief without the CNS liabilities associated with CB_1_ modulation.^24,25^ As a result, treatments like olorinab that selectively target CB_2_ receptors and are peripherally active may offer a rational approach to pain management.

Treatment of abdominal pain is a notable unmet need for patients with CD. Over-the-counter drugs, such as NSAIDs, are often the first-line treatment for abdominal pain in CD. However, NSAIDs, although effective for pain relief, are associated with an increased risk of gut mucosal damage, ulceration, and induction of irritable bowel disease flares.^6^ Therefore, further pain management is often needed. Opioids are frequently used and effective for treating severe acute pain in CD. However, concerns with opioid use include tolerance development, addiction and abuse, constipation, narcotic bowel syndrome, increased susceptibility to infection, and respiratory depression and death.^5,26^ Antispasmodics and antidepressants, which are often used as an “adjuvant analgesics” to chronic opioid therapy, are supported by limited evidence demonstrating pain reduction in patients with CD.^4,26^

Cannabis has been shown to induce a clinical symptom response in patients with CD in a prospective placebo-controlled study (based on the CD Activity Index)^8^ and in a retrospective observational study (based on the Harvey–Bradshaw Index).^9^ Frequent cannabis use in CD patients, largely for abdominal pain, nausea, and decreased appetite, has also been reported.^27^ However, cannabis consists of a poorly reproducible mixture of hundreds of pharmacologically active compounds, which vary with the strain, season, and maturity of plants.^28^ Therefore, a substantial amount of variation in potency and efficacy are to be expected, as well as off-target adverse effects on cognitive function limiting its use.^28^

In this population of subjects who experienced continued abdominal pain despite remission or mildly active inflammation, selective and full agonism of CB_2_ with olorinab was associated with rapid onset of action and sustained pain response throughout the 8 weeks of treatment. Importantly, this group of subjects experienced pain for at least 7 consecutive days during the screening period, and during treatment, subjects experienced more pain-free days over time. In addition, no pain medication was required by any subject during the study, which further supports the pain relief evinced by improved average pain scores.

All subjects in this study had quiescent to mildly active CD at baseline. No significant changes in CRP or fecal calprotectin were observed with olorinab at the doses used, consistent with previous trials of cannabis or cannabidiol-rich botanical extract for ulcerative colitis that did not find a statistically significant effect on CRP, fecal calprotectin, or other disease-specific markers.^29,30^ This suggests that any effects on pain reduction may be independent of changes in intestinal inflammation. However, larger trials including patients with a higher inflammatory burden are required to confirm whether or not olorinab has any impact on inflammatory markers.

This proof-of-concept study demonstrated encouraging results but was limited by a small sample size, the open-label study design, and the lack of a placebo control treatment group. Larger studies of olorinab for gastrointestinal-related pain are warranted.

## CONCLUSIONS

The lack of nonaddicting medications with favorable safety profiles for the treatment of abdominal pain in CD is an unmet medical need. This phase 2a study demonstrated that olorinab was well tolerated and provided preliminary evidence of efficacy in the treatment of abdominal pain associated with CD. These data support the continued clinical development of olorinab for the management of abdominal pain associated with CD and potentially other gastrointestinal diseases.

## Supplementary Material

otaa089_suppl_Supplementary_Materials_1

## Notes

Funding: This study was supported by Arena Pharmaceuticals, Inc., San Diego, CA.

Conflict of Interest: B.R. Yacyshyn has received research funding from Arena Pharmaceuticals, Merck, and Procter & Gamble Pharmaceuticals; and has received consulting fees from Arena Pharmaceuticals, Gilead Sciences, and Procter & Gamble Pharmaceuticals. S. Hanauer has served as a consultant and advisor for Arena Pharmaceuticals. P. Klassen, B.A. English, K. Stauber, K. Gilder, and S. Turner have ownership interest in and are employees of Arena Pharmaceuticals. P.D.R. Higgins has received research funding from AbbVie, Arena Pharmaceuticals, the Crohn’s & Colitis Foundation, Eli Lilly, Genentech, Janssen, the National Institutes of Health, Pfizer, Roche, Shire Pharmaceuticals, Seres Therapeutics, TARGET PharmaSolutions, Takeda, and the University of California, Berkeley; has received consulting fees from AbbVie, Arena Pharmaceuticals, Eli Lilly, Pfizer, PRIME Medical Education, and Takeda; and has participated in speakers’ bureaus for the GI Health Foundation. C.F. Barish has nothing to disclose.
