# Supplementary material for: Safety, Pharmacokinetics, and Efficacy of Olorinab, a Peripherally Acting, Highly Selective, Full Agonist of the Cannabinoid Receptor 2, in a Phase 2a Study of Patients With Chronic Abdominal Pain Associated With Crohn’s Disease
Source: Crohns Colitis 360. 2020 Oct 24;3(1):otaa089. doi: 10.1093/crocol/otaa089 (PMC9802051; doi:10.1093/crocol/otaa089)
Supplement: otaa089_suppl_Supplementary_Materials_1 [file otaa089_suppl_supplementary_materials_1.docx]

**List of Supplemental Digital Content for “Safety, Pharmacokinetics, and Efficacy of Olorinab, a Peripherally Acting, Highly Selective, Full Agonist of the Cannabinoid Receptor 2, in a Phase 2a Study of Patients With Chronic Abdominal Pain Associated With Crohn’s Disease”**

| **Supplementary Table 1.** Pharmacokinetic Parameters for Olorinab and  its Metabolites. | Page 2-5 |
| --- | --- |
| **Supplementary Table 2.** Ratio of Olorinab Metabolites to Olorinab Using Pharmacokinetic Parameters. | Page 6-7 |
| **Supplementary Table 3.** Percentage Change From Baseline to Week 8 in CD-PRO Domain Scores. | Page 8 |
| **Supplementary Figure 1.** Pain Relief Response With Olorinab. | Page 9-11 |
| **Supplementary Figure 2.** C-Reactive Protein and Fecal Calprotectin Levels. | Page 12-13 |

**SUPPLEMENTARY APPENDIX**

**Supplementary Table 1. Pharmacokinetic Parameters for Olorinab and its Metabolites**

| **Analyte** | **Timepoint** | **Treatment** | **C_max_,  geometric mean  (%CV),  ng/mL** | **t_max_,  median  (min, max), h** | **AUC_0-8_,  geometric mean  (%CV),**  **ng·h/mL** |
| --- | --- | --- | --- | --- | --- |
| Olorinab | Week 0 | 25 mg  single dose | n = 6 | n = 6 | n = 6 |
|  |  |  | 409 (59.2) | 2.02 (1.00, 4.12) | 1690 (54.0) |
|  |  | 100 mg  single dose | n = 8 | n = 8 | n = 8 |
|  |  |  | 1800 (55.6) | 2.00 (0.57, 2.12) | 6890 (39.4) |
|  | Week 8 | 25 mg TID | n = 5 | n = 5 | n = 4 |
|  |  |  | 624 (71.2) | 1.00 (1.00, 4.03) | 2280 (124.2) |
|  |  | 100 mg TID | n = 4 | n = 4 | n = 4 |
|  |  |  | 2280 (34.1) | 2.00 (1.03, 2.00) | 9010 (41.4) |
|  | Accumulation Ratio^a^ | 25 mg TID | n = 5 | - | n = 4 |
|  |  |  | 1.53 (40.1) | - | 1.41 (42.6) |
|  |  | 100 mg TID | n = 4 | - | n = 4 |
|  |  |  | 1.08 (41.4) | - | 1.16 (9.9) |
| M1 | Week 0 | 25 mg  single dose | n = 6 | n = 6 | n = 6 |
|  |  |  | 171 (36.4) | 3.02 (1.00, 8.00) | 823 (39.4) |
|  |  | 100 mg  single dose | n = 8 | n = 8 | n = 8 |
|  |  |  | 712 (82.8) | 3.00 (1.08, 4.15) | 3560 (68.4) |
|  | Week 8 | 25 mg TID | n = 5 | n = 5 | n = 4 |
|  |  |  | 228 (57.0) | 2.00 (2.00, 3.87) | 1380 (74.4) |
|  |  | 100 mg TID | n = 4 | n = 4 | n = 4 |
|  |  |  | 1340 (45.9) | 2.99 (2.00, 4.02) | 7410 (41.4) |
|  | Accumulation ratio^a^ | 25 mg TID | n = 5 | - | n = 4 |
|  |  |  | 1.29 (47.2) | - | 1.62 (35.3) |
|  |  | 100 mg TID | n = 4 | - | n = 4 |
|  |  |  | 1.19 (38.4) | - | 1.33 (23.7) |
| M2^b^ | Week 0 | 25 mg  single dose | n = 6 | n = 6 | n = 6 |
|  |  |  | 15.3 (129.4) | 8.00 (6.17, 8.22) | 55.9 (103.7) |
|  |  | 100 mg  single dose | n = 8 | n = 8 | n = 8 |
|  |  |  | 29.8 (100.3) | 8.01 (4.15, 8.27) | 112 (104.5) |
|  | Week 8 | 25 mg TID | n = 5 | n = 5 | n = 4 |
|  |  |  | 87.3 (124.4) | 0.00 (0.00, 5.93) | 875 (70.0) |
|  |  | 100 mg TID | n = 4 | n = 4 | n = 4 |
|  |  |  | 278 (48.0) | 5.05 (0.00, 8.00) | 1570 (82.1) |
| M4^b^ | Week 0 | 25 mg  single dose | n = 6 | n = 6 | n = 6 |
|  |  |  | 20.9 (176.7) | 8.03 (2.00, 8.22) | 68.0 (185.8) |
|  |  | 100 mg  single dose | n = 8 | n = 8 | n = 8 |
|  |  |  | 26.4 (81.1) | 7.02 (2.00, 8.12) | 114 (65.8) |
|  | Week 8 | 25 mg TID | n = 5 | n = 5 | n = 4 |
|  |  |  | 90.4 (91.4) | 5.93 (0.00, 6.13) | 809 (83.7) |
|  |  | 100 mg TID | n = 4 | n = 4 | n = 4 |
|  |  |  | 209 (77.3) | 3.06 (0.00, 8.00) | 1150 (100.9) |

AUC_0-8_, area under the concentration-time curve from 0 to 8 hours postdose; C_max_, maximum (peak) observed plasma concentration; CV, coefficient of variation; PK, pharmacokinetics; TID, three times daily; t_max_, time to reach maximum (peak) observed plasma concentration.

^a^Accumulation ratio = Week 8 vs Week 0. Accumulation ratio only reported for olorinab and M1.

^b^Values for C_max_, t_max_, and AUC_0-8_ should be interpreted with caution due to the slow rate of formation for these metabolites and the PK sampling schedule used in this study, which likely underestimated these values.

**Supplementary Table 2. Ratio of Olorinab Metabolites to Olorinab Using Pharmacokinetic Parameters at Week 8**

| **Analyte** | **Treatment** | **Metabolite to Parent Ratio** | |
| --- | --- | --- | --- |
|  |  | **C_max_,  geometric mean  (%CV)** | **AUC_0-8_,  geometric mean  (%CV)** |
| M1 | 25 mg TID | n = 5 | n = 5 |
|  |  | 0.365 (44.0) | 0.603 (34.7) |
|  | 100 mg TID | n = 4 | n = 4 |
|  |  | 0.586 (37.6) | 0.822 (40.8) |
| M2^a^ | 25 mg TID | n = 5 | n = 4 |
|  |  | 0.140 (110.3) | 0.384 (65.1) |
|  | 100 mg TID | n = 4 | n = 4 |
|  |  | 0.122 (34.7) | 0.174 (86.0) |
| M4^a^ | 25 mg TID | n = 5 | n = 4 |
|  |  | 0.145 (72.9) | 0.355 (54.5) |
|  | 100 mg TID | n = 4 | n = 4 |
|  |  | 0.0915 (55.8) | 0.128 (81.4) |

AUC_0-8_, area under the concentration-time curve from 0 to 8 hours postdose; C_max_, maximum (peak) observed plasma concentration; CV, coefficient of variation; PK, pharmacokinetics; TID, three times daily.

^a^Values for C_max_ and AUC_0-8_ should be interpreted with caution due to the slow rate of formation for these metabolites and the PK sampling schedule used in this study, which likely underestimated these values.

**Supplementary Table 3.** **Percentage Change From Baseline to Week 8 in CD-PRO Domain Scores**

| **Treatment Group** | **Domain** | **n** | **Percentage Change From Baseline, mean (SD)** | ***P* value vs Baseline^a^** |
| --- | --- | --- | --- | --- |
| 25 mg TID | Bowel | 6 | −34.5 (30.5) | 0.0390 |
|  | Abdominal Function | 5 | −32.5 (29.6) | 0.0698 |
|  | Systemic Symptoms | 6 | −49.3 (35.1) | 0.0185 |
|  | Emotional | 5 | −46.8 (30.3) | 0.0261 |
|  | Daily Impact | 4 | −58.3 (33.7) | 0.0407 |
|  | Coping | 4 | −50.0 (13.6) | 0.0052 |
| 100 mg TID | Bowel | 6 | −47.7 (29.4) | 0.0107 |
|  | Abdominal Function | 6 | −26.3 (19.3) | 0.0207 |
|  | Systemic Symptoms | 6 | 3.37 (47.7) | 0.8691 |
|  | Emotional | 6 | −23.5 (59.6) | 0.3779 |
|  | Daily Impact | 6 | −26.0 (60.7) | 0.3420 |
|  | Coping | 6 | −38.3 (54.9) | 0.1480 |

CD-PRO, Crohn’s disease patient-reported outcomes; SD, standard deviation; TID, three times daily.

^a^One-sample *t* test.

**Supplementary Figure 1. Pain Relief Response With Olorinab.** Proportion of patients who had ≥30% reduction from baseline in weekly peak AAPS (**A**), trough AAPS (**B**), evening trough AAPS (**C**), and daily AAPS (averaged over peak, trough, and evening trough diary entries; **D**) with olorinab 25 mg TID or 100 mg TID and in all subjects at Weeks 1, 2, 4, 6, and 8, and were considered pain relief responders. Pain relief response was assessed using non-responder imputation (i.e., subjects who withdrew early or did not have Week 8 data were considered non-responders). EOT analysis used the last available value for subjects who withdrew early or did not have Week 8 data. AAPS, average abdominal pain score; EOT, end of treatment; TID, three times daily.

| **A** |
| --- |
| 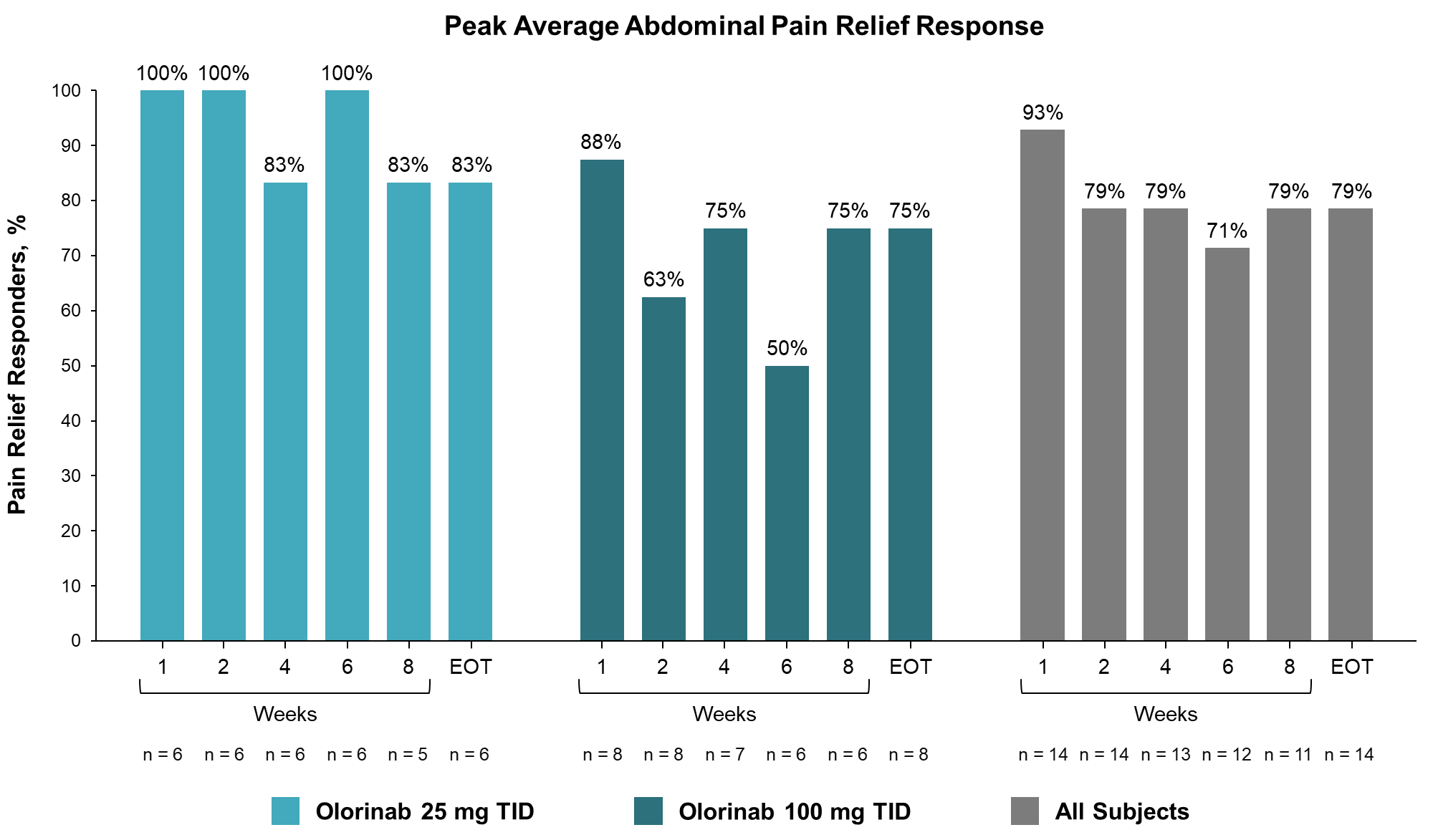 |
| **B** |
| 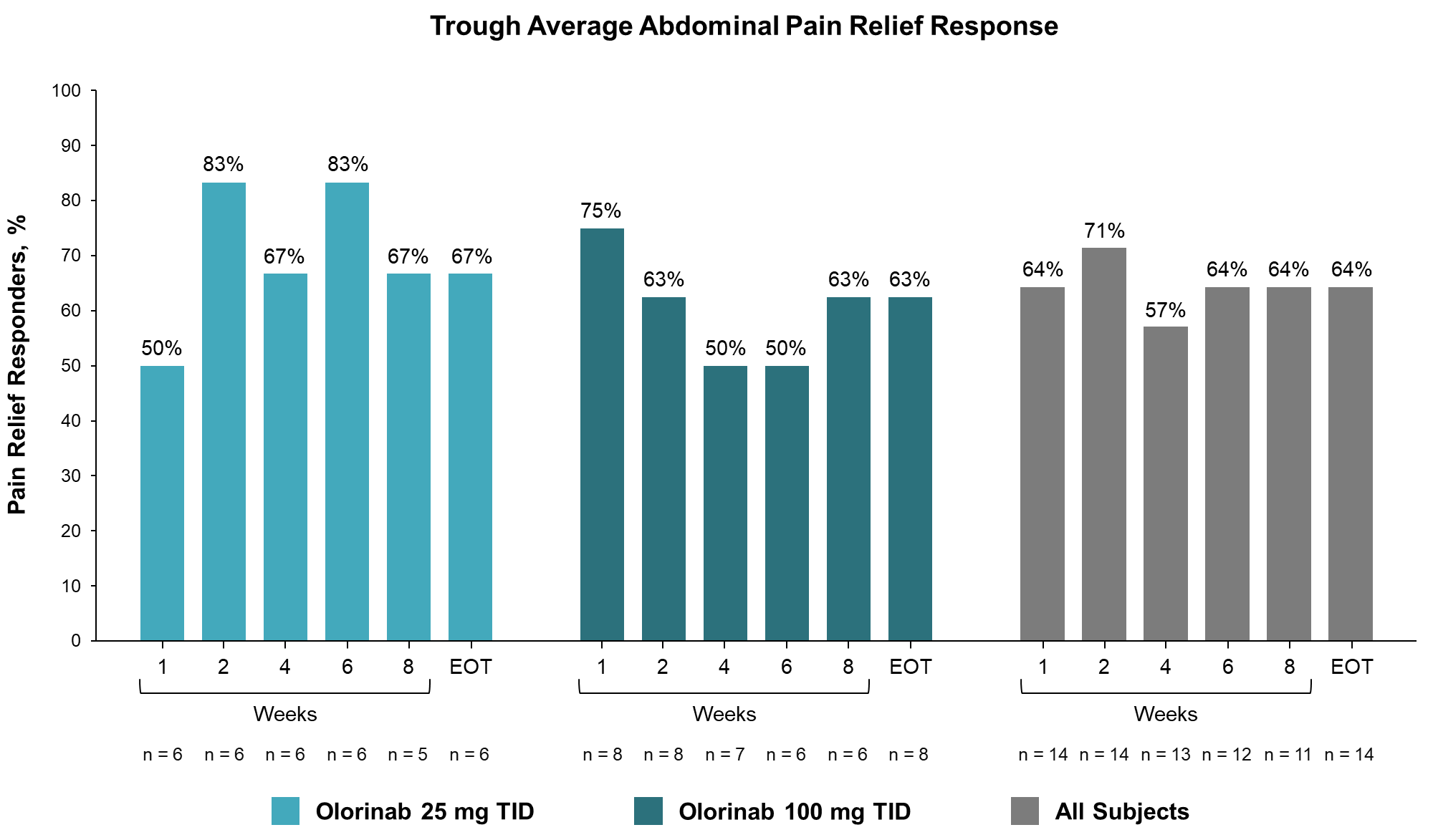 |
| **C** |
| 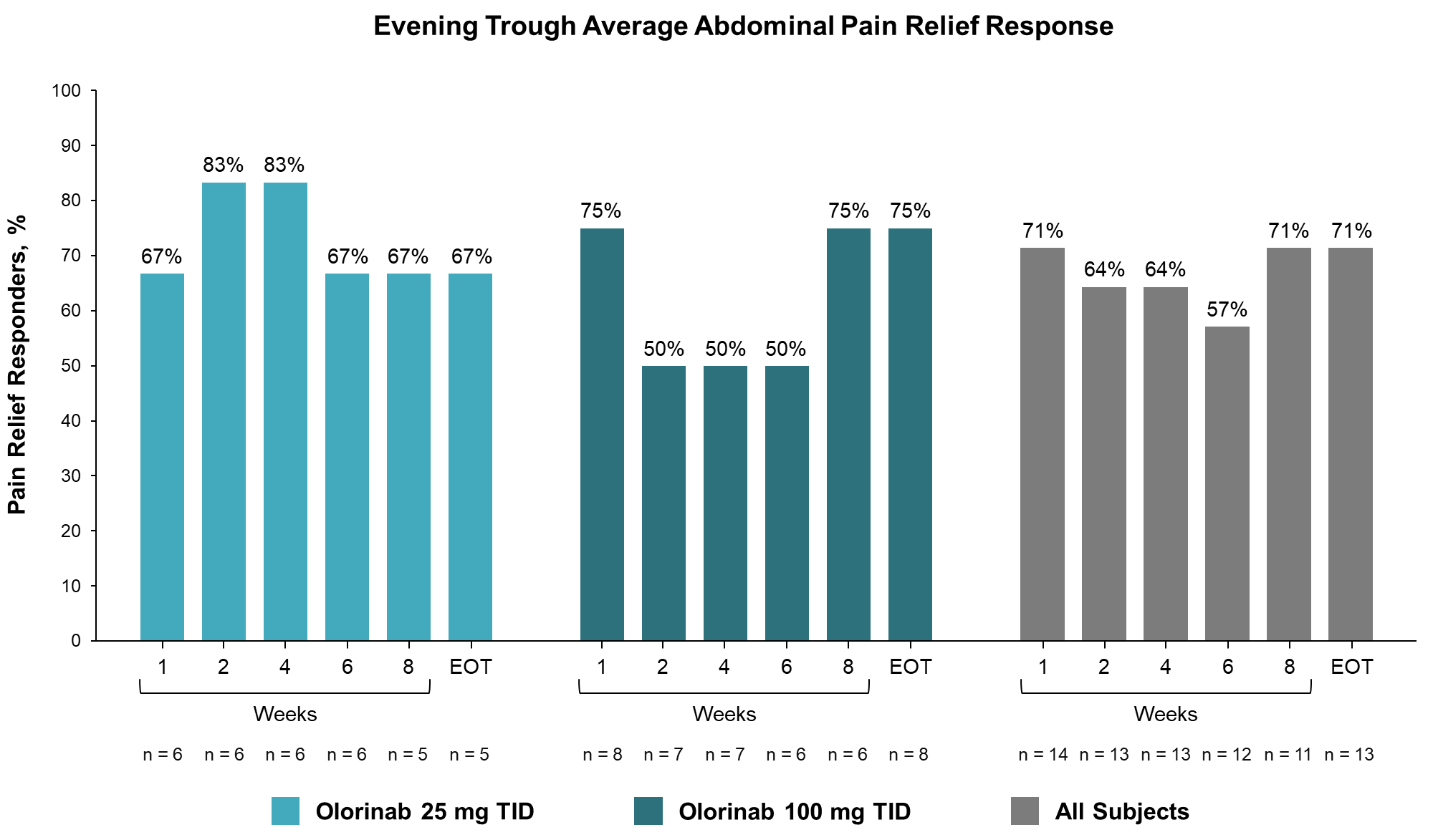 |
| **D** |
| 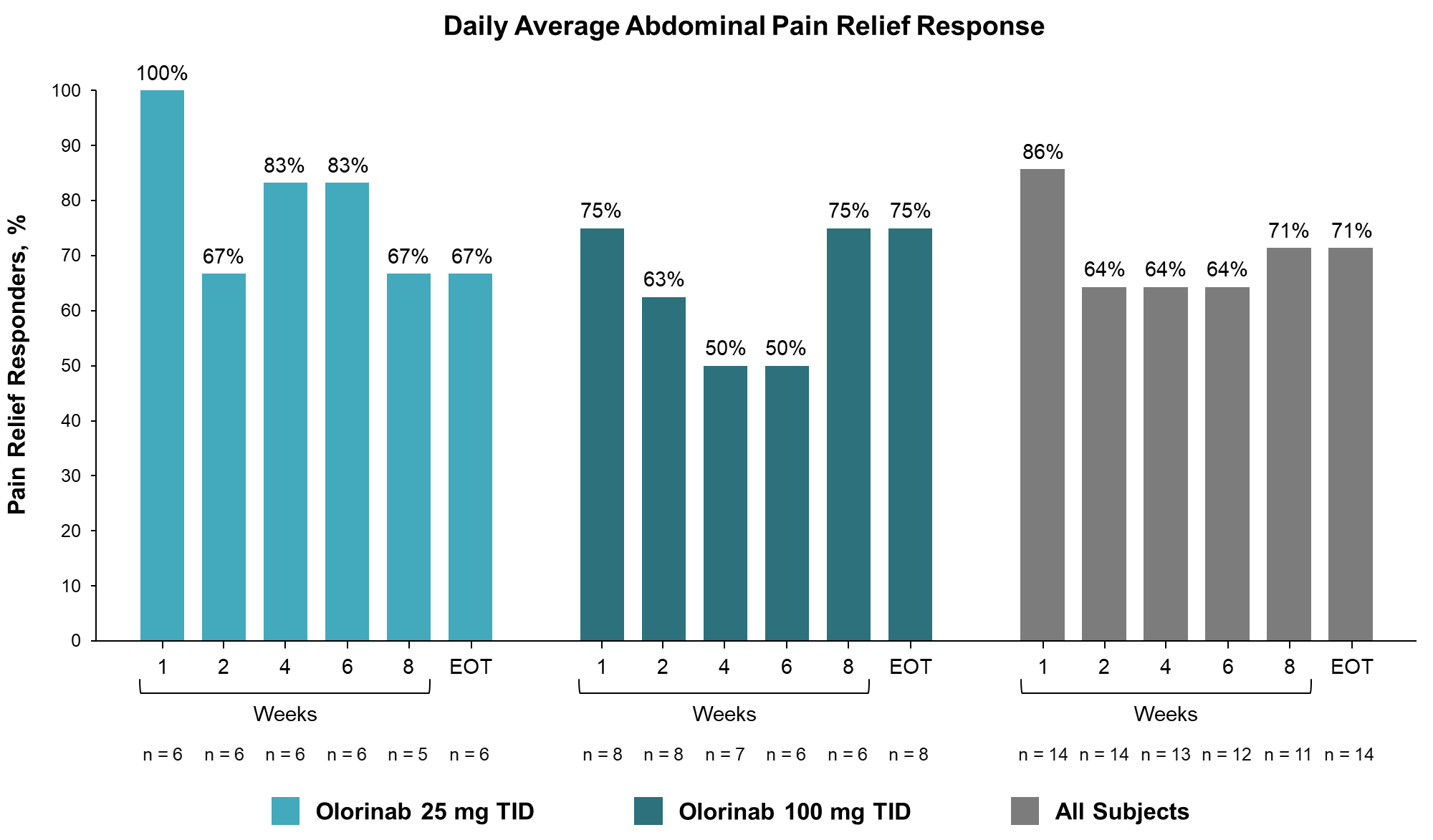 |

**Supplementary Figure 2. C-Reactive Protein and Fecal Calprotectin Levels.** The mean (SD) concentration of C-reactive protein (**A**) and fecal calprotectin (**B**) measured at Baseline, Week 4, and Week 8 visits in patients receiving olorinab 25 mg TID or 100 mg TID. BL, baseline; SD, standard deviation; TID, three times daily.

| **A** |
| --- |
| 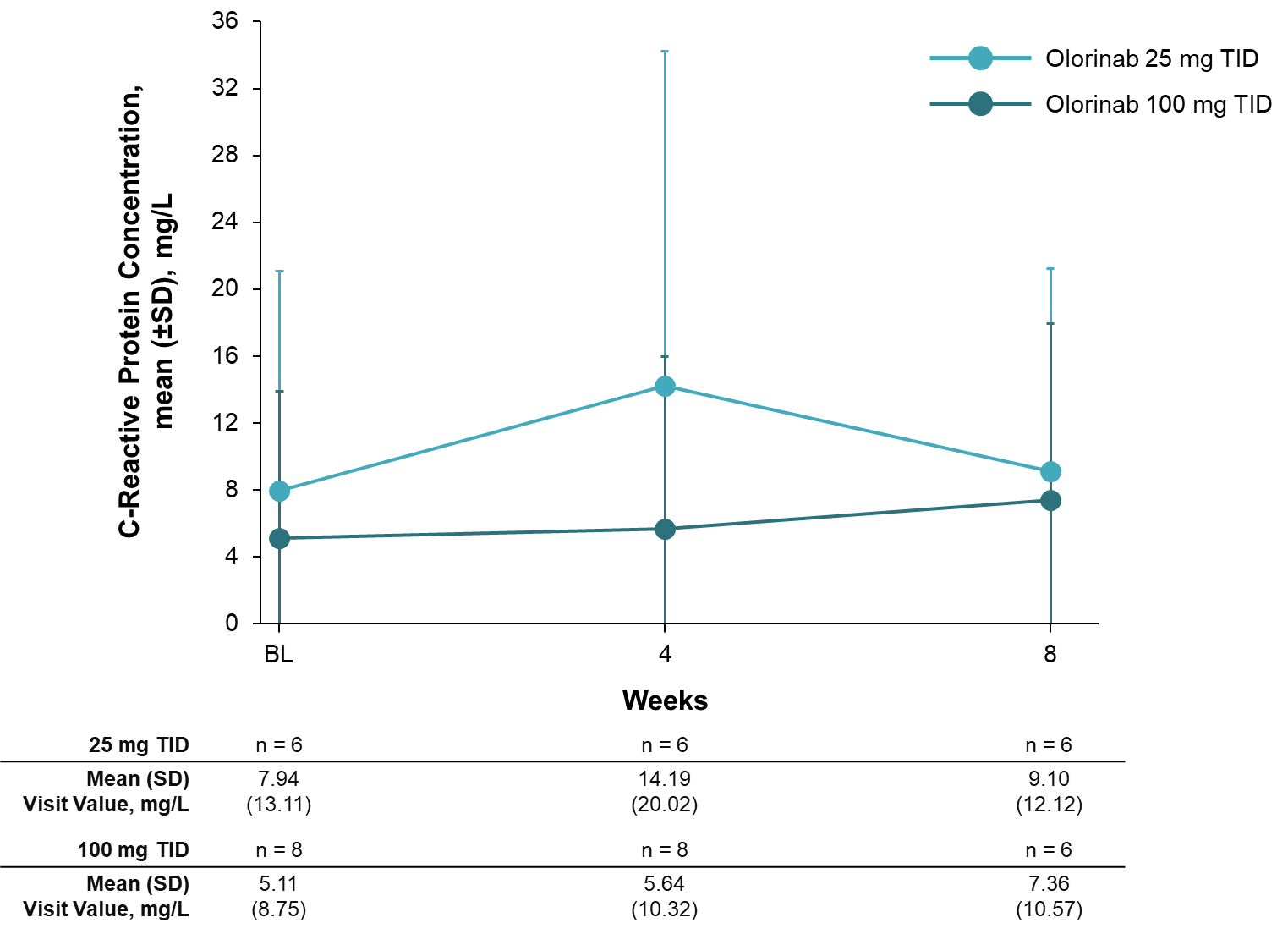 |
| **B** |
| 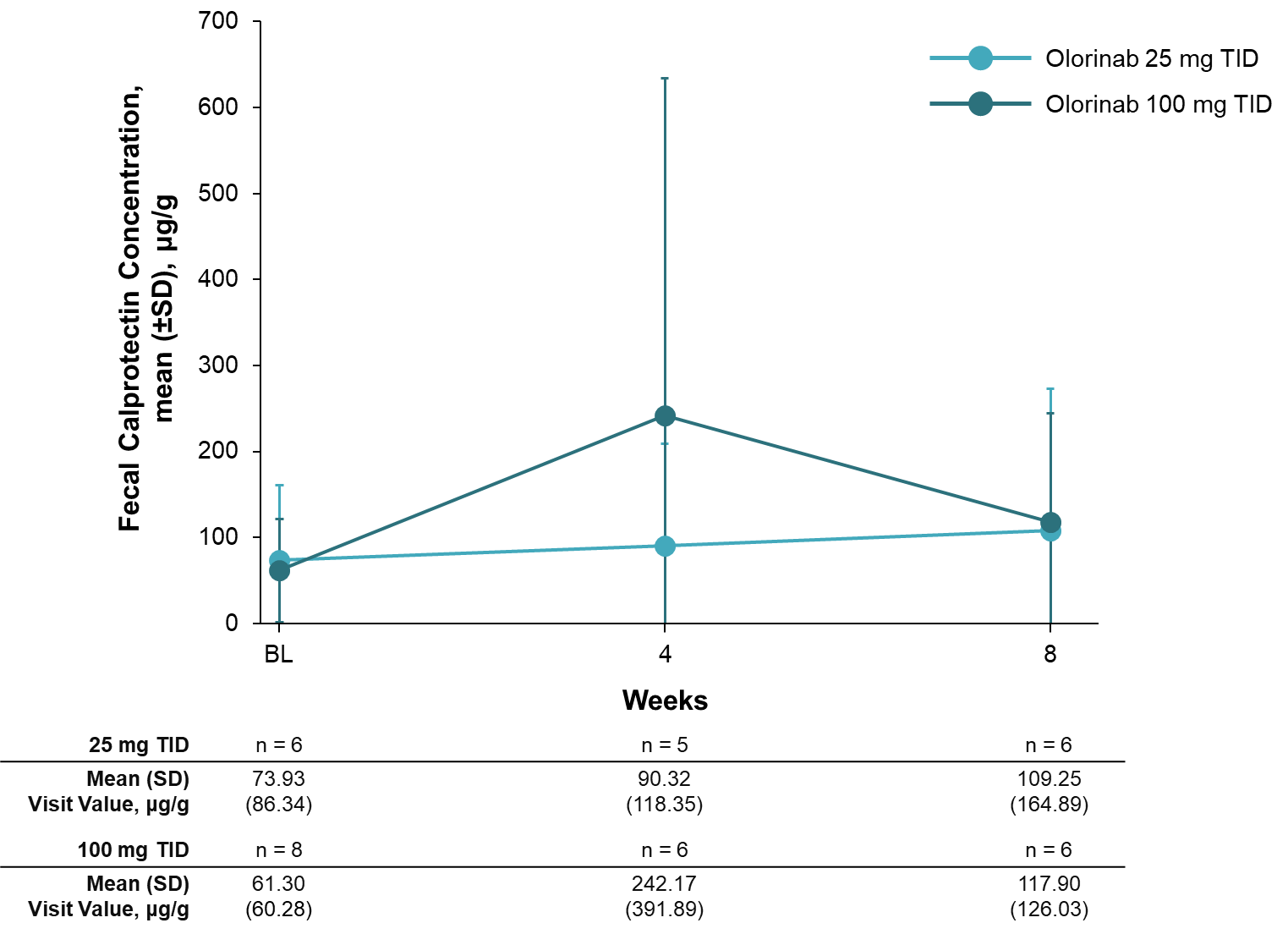 |
